# Phase Resetting Reveals Network Dynamics Underlying a Bacterial Cell Cycle

**DOI:** 10.1371/journal.pcbi.1002778

**Published:** 2012-11-29

**Authors:** Yihan Lin, Ying Li, Sean Crosson, Aaron R. Dinner, Norbert F. Scherer

**Affiliations:** 1 Department of Chemistry, University of Chicago, Chicago, Illinois, United States of America; 2 Institute for Biophysical Dynamics, University of Chicago, Chicago, Illinois, United States of America; 3 Department of Physics, University of Chicago, Chicago, Illinois, United States of America; 4 Department of Biochemistry and Molecular Biology, University of Chicago, Chicago, Illinois, United States of America; Princeton University, United States of America

## Abstract

Genomic and proteomic methods yield networks of biological regulatory interactions but do not provide direct insight into how those interactions are organized into functional modules, or how information flows from one module to another. In this work we introduce an approach that provides this complementary information and apply it to the bacterium *Caulobacter crescentus*, a paradigm for cell-cycle control. Operationally, we use an inducible promoter to express the essential transcriptional regulatory gene *ctrA* in a periodic, pulsed fashion. This chemical perturbation causes the population of cells to divide synchronously, and we use the resulting advance or delay of the division times of single cells to construct a phase resetting curve. We find that delay is strongly favored over advance. This finding is surprising since it does not follow from the temporal expression profile of CtrA and, in turn, simulations of existing network models. We propose a phenomenological model that suggests that the cell-cycle network comprises two distinct functional modules that oscillate autonomously and couple in a highly asymmetric fashion. These features collectively provide a new mechanism for tight temporal control of the cell cycle in *C. crescentus*. We discuss how the procedure can serve as the basis for a general approach for probing network dynamics, which we term chemical perturbation spectroscopy (CPS).

## Author Summary

During the cell cycle, the cell progresses through a series of stages that are associated with various cell cycle events such as replication of genetic materials. Genetic and molecular dissections have revealed that the cell cycle is regulated by a network of interacting molecules that produces oscillatory dynamics. The major cell cycle regulators have been identified previously in different species and the activity of these regulators oscillates. However, the question of how cell cycle regulators coordinate different cell cycle events during the cell cycle remains controversial. Here, we investigate this question in a model bacterial system for cell cycle, *Caulobacter crescentus*. We perturb the expression of the master cell cycle regulator *ctrA* in a pulsatile fashion and quantify the response of the cell cycle to such perturbations. The measured response is contradictory to the existing mechanism of *Caulobacter* cell cycle control, which views the cell cycle progression as a sequential activation/inhibition process. We propose a new model that involves coupling of multiple oscillators and show the quantitative agreement between this new model and our measurements. We expect this procedure to be generalized and applied to a broad range of systems to obtain information that complements that obtained from other methods.

## Introduction

The regulatory network that coordinates oscillating periods of growth, chromosome replication, and division is among the most important in a cell [1]. It is emerging that the cell cycle network, like others, is organized into functional modules [1]–[5]. Each module is sequentially activated or inhibited by key cell cycle regulatory proteins, whose concentrations oscillate with the same period to ensure irreversibility and a “once-per-cell-cycle” occurrence of each process [1], [2]. However, in both prokaryotes and eukaryotes there is increasing evidence that internal regulatory modules (i.e., a set of chemical reactions associated with a key sub-function of the overall cell cycle) can run autonomously. For example, in the bacterium *C. crescentus* several rounds of chromosome replication can occur under conditions where activity of the master cell cycle regulator CtrA is largely suppressed [6], and certain *C. crescentus* mutants can undergo multiple cell constrictions within one cell cycle [7]–[9]. In budding yeast, cell cycle modules such as budding [10], transcription [11], centrosome replication [12], and Cdc14 localization [4], [13] can run independently of Cdk activity. This raises the question of how individual modules interact to generate robust sequences of events.

The interactions defining the connectivity of a regulatory network, such as that controlling the cell cycle, can be dissected in a traditional manner by functional reconstitution [14]. However, this does not provide information about the integrated dynamics of the interacting network as a whole. Alternatively, by applying appropriate perturbations to an intact network, one can determine the dynamics of the response of one or more measureable parameters and infer global properties of the network that underlie a given process. We refer to this as probing the topology of the functional relations of the network. Such an approach is analogous to circuit analysis in electrical engineering and time-resolved spectroscopies employed in chemistry and physics [15], [16]. Here we report a periodic perturbation approach that provides insight into the systems-level control features of a bacterial cell cycle.

**Figure 1:**
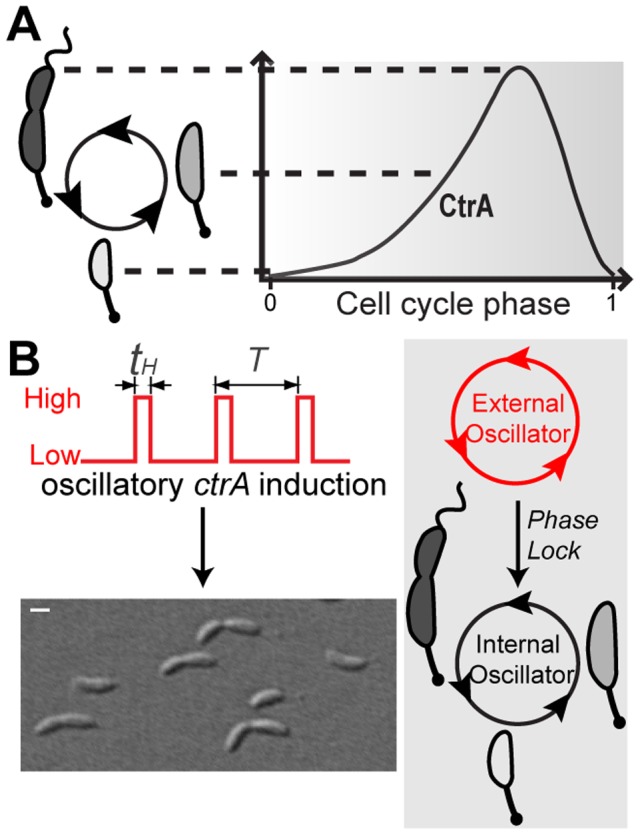
Schematic for phase locking the stalked *C. crescentus* cell division cycle by periodically perturbing *ctrA* expression. (**A**) *C. crescentus* stalked cell cycle is driven by oscillating concentration of the master regulator protein, CtrA. The cell cycle begins with low CtrA concentration, allowing initiation of chromosome replication. CtrA levels then rise gradually, accompanied by cell growth and division. Cytoplasmic compartmentalization at the pre-divisional stage triggers the rapid proteolysis of CtrA, initiating another round of stalked cell division. (**B**) Schematic of phase locking. (Left) The expression of exogenous *ctrA* (in a mutant lacking endogenous *ctrA*) is controlled by a periodic inducer pulse train which oscillates between two discrete levels (Low and High), which then phase locks the dividing stalked cells on the surface of a microfludic flow channel (lower micrograph) as schematized on the right.

Specifically, we study *Caulobacter crescentus* because its cell cycle regulatory network has been well-characterized both genetically and biochemically [17] and quantitative models have been reported [18]–[20]. The life cycle of *C. crescentus* begins as a non-reproductive motile swarmer cell, with chromosome replication inhibited by the cell cycle master regulator CtrA [21] binding to the replication origin [6]. The *C. crescentus* swarmer cell then differentiates into a reproductive sessile stalked cell (i.e., the mother); this cell differentiation event is concomitant with proteolytic clearing of CtrA from the cell. The stalked cell then commences DNA replication, cell growth, FtsZ ring formation, and membrane fission to yield a daughter swarmer cell and regenerate the mother stalked cell [22] (**Figure 1A** left). While swarmer progeny remain in a gap-like phase prior to differentiation, the stalked progeny can continue to reproduce for tens of generations [20], [23]. Thus the stalked cell behaves as a self-sustained oscillator. The CtrA concentration profile during the stalked cell cycle, shown schematically in **Figure 1A**, is low in the early stalked cell, reaches a maximum at the late-predivisional stage, then decreases rapidly in the stalked compartment (post-constriction) prior to initiation of a successive round of reproduction [17], [24], [25]

The design of our experiment is as follows (**Figure 1B**). In contrast to knockout experiments that completely eliminate an element of a regulatory network, we seek to quantitatively perturb the expression of a molecule and analyze the resulting change in system dynamics. To this end, we engineer a mutant strain that lacks *ctrA* and then introduce a xylose-inducible *ctrA* on a plasmid [26]. It is important to note that this strain (with the plasmid) grows and divides in an essentially normal fashion in the presence of constant xylose concentration (≥0.9×10^−4^%, w/v; Figure S1 in Text S1). This is made possible by the fact that the active form of CtrA is the phosphorylated protein [24], [25], which can still oscillate even though the gene is transcribed at a constant rate. We then use a microfluidic device to toggle between low and high levels of xylose. Although the inducer concentrations are such that the protein should always be present at levels that permit division, the periodic pulses of expression must indirectly increase the amount of phosphorylated protein because they cause division to synchronize. We measure division times of single cells and use them to determine the advance or delay of the cell cycle as a function of its phase when a pulse arrives. This response defines the phase resetting curve (PRC), which informs a mathematical analysis that reveals two important insights into the cell-cycle network: (i) it comprises functional modules that oscillate autonomously and (ii) the coupling between these units is highly asymmetric such that CtrA acts to brake rather than drive the cell cycle. We validate this model by quantitative comparison with independent experimental data. We discuss molecular mechanisms for realizing the elucidated functional features and their potential biological advantages.

## Results/Discussion

### Validation of the experimental construct

As discussed in the Introduction, we examine the quantitative change in division times in response to pulses in CtrA in a *C. crescentus ctrA* mutant strain with a xylose-inducible copy of *ctrA*. Unless otherwise indicated, we switch between xylose levels of 0.9×10^−4^% (w/v) and 0.03%. To ensure that CtrA was not limiting, we first measured inter-division times for *fixed* xylose levels. The strain is viable for xylose concentrations ≥0.9×10^−4^%. The mean inter-division time at 0.9×10^−4^% xylose is 68.1±15.6 min (N = 5160 cell division events; temperature = 32.0°C), which is comparable to the wild-type, although it should be noted that the noise (standard deviation/mean) is larger (**Figure S1** in Text S1).

**Figure 2:**
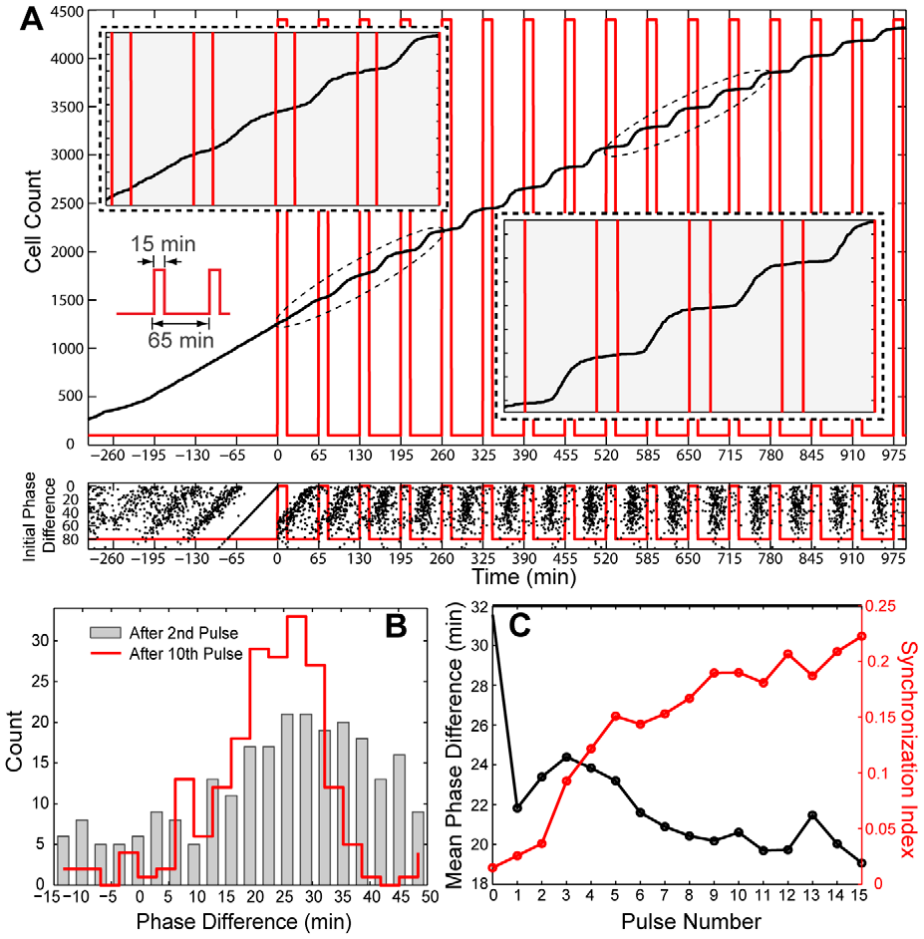
*C. crescentus* cells can be phase locked. (**A**) Phase locking a population of single cells. The upper panel shows the cell growth trajectory overlapped with the external inducer pulse train (**Text S1**). The inserts are magnified views from 0 to 260 min and from 520 to 780 min. The lower panel shows the divisions of single cells (261 cells at pulse start) that were monitored for over 20 hrs. The timing of division events for individual cells are plotted (black dots) along lines parallel to the Time axis. Cells are arranged along the vertical axis according to their phases prior to the first perturbation (i.e., the diagonal line immediately before time zero). Inducer profile along experimental time is indicated in red, where high and low xylose levels are 0.03% (w/v) and 0.00009% (w/v) respectively. (**B**) Phase difference distribution. Phase difference (in minutes) between the internal cell cycle oscillator and external oscillator is analyzed. The distributions of phase difference after 2^nd^ and 10^th^ pulses are shown. (**C**) Quantification of phase evolution and division synchrony. The distributions in (b) are used to quantify the mean phase difference and synchronization. Both quantities are plotted with respect to the number of pulses delivered.

In the periodic experiment, we stimulate a population of surface attached stalked cells cultured in a Y-shaped microfluidic device [20] with a pulse train that alternates between low and high xylose concentrations (**Figure 1B** left, **Methods**). We explored a range of external pulse periods that was centered about the mean intrinsic cell-cycle time (i.e., 68.1 min). **Figure 2** shows the results obtained with pulses of 15 min high xylose (0.03%) and 50 min low xylose concentration (0.9×10^−4^%) (i.e., external oscillator period is 65 min). The entrainment of the cell cycle to this external periodic pulse train can be readily visualized in the growth curve that is constructed from the measured single cell divisions, only counting progeny of the original stalked cells (**Figure 2A** upper panel; **Methods**). The linear growth of the initial portion of the curve is due to asynchronous division, and the subsequent stepwise growth corresponds to synchronous division [27]. Similar synchronization was recently realized for synthetic genetic oscillators [28]. Here, the synchronization confirms that the pulses in (initially non-phosphorylated and thus inactive) CtrA are sufficient to perturb the cell cycle and serve as the basis for phase resetting. This observation is consistent with the idea that the active form of CtrA follows the overall CtrA protein level closely owing to rapid phosphorylation during the stalked cell cycle [24], [25], [29], [30].

### The phase resetting curve is sufficient to capture the statistics of division

Many (∼20) divisions were followed for each cell. Each division event is indicated by a dot in **Figure 2A** (lower panel), with the timing of the event on the horizontal axis and its lineage on the vertical axis (labeling each original stalked cell by its cell-cycle phase immediately prior to the first pulse). By construction, the initial condition is a diagonal line in this representation; it reflects an asynchronous population with a large dispersion in phase over the cell cycle immediately before perturbation. The dispersion from a line for divisions at negative time in this reference frame (i.e., reading to the left in **Figure 2A**) shows the intrinsic noise in the cell cycle. After the pulse train starts (positive time), the population gradually evolves to a distribution about a vertical line, indicating synchronous division. The distribution narrows and the synchronization, as quantified by an information theoretic measure, the synchronization index [31] (see **Methods**), increases as more pulses are delivered (**Figs. 2B and 2C**). Thus quantitative metrics support phase-locking of the cell cycle.

**Figure 3:**
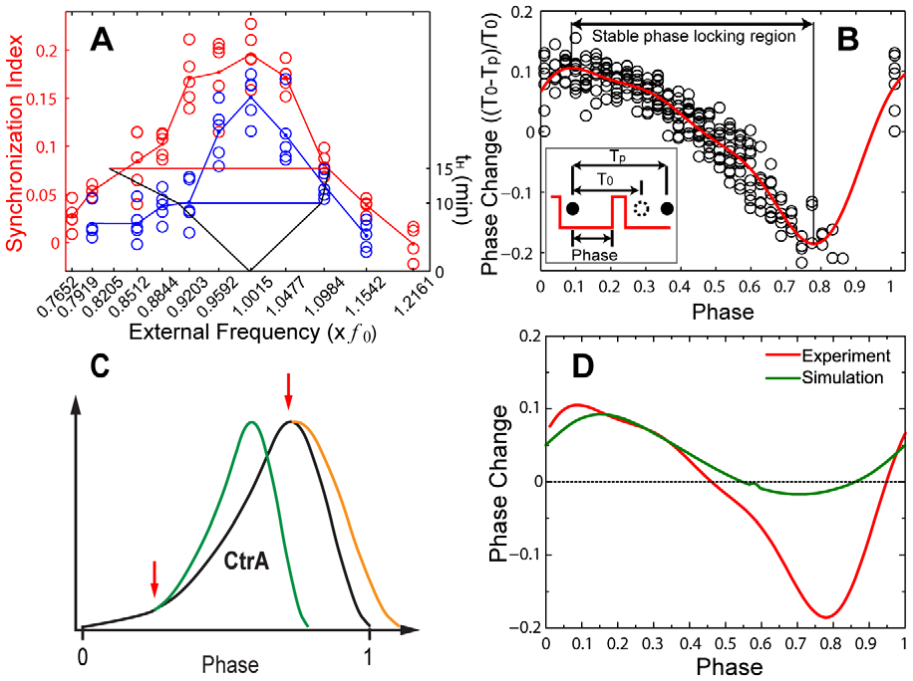
The phase is more readily delayed than advanced. (**A**) Quantification of synchronization index under various external pulse profiles. The synchronization index ranges from zero to one as the population varies from asynchronous to synchronous. The synchronization indices (less the initial value) from the eighth to twelfth pulses are plotted for a variety of external periods ranging from 56 min to 89 min (converted to frequency) with 10 min and 15 min pulses (left vertical axis). The horizontal bars (right vertical axis) indicate the range for 1∶1 phase locking of a noise-free cell cycle oscillator (Arnold Tongues). Such frequency ranges are inferred from the phase resetting curves in (a) and (b). 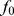 is the intrinsic frequency. (**B**) Phase resetting curve (PRC) for 15 min pulses. The data (open circles) are fitted with a real trigonometric polynomial of degree three (solid line) to ensure periodicity. (**C**) Schematics for perturbations on CtrA oscillation by a single elevated *ctrA* expression pulse at two possible time points. (**D**) Comparison between experimental and simulated 15-min-pulse PRCs based on the model of Li and Tyson [19].

The phase-locking efficacy varies across the range of external pulse frequencies (i.e., inverse periods) explored (**Figure 3A** **and Figure S5** in Text S1) owing to the intrinsic noise in the cell cycle. Stronger entrainment occurs when *ctrA* is induced for 15 min than 10 min for the same overall pulse frequency. The synchronization index peaks when the pulse frequency equals the intrinsic cell-cycle frequency and decays more rapidly at higher frequencies than at lower frequencies. The asymmetry of the response can be seen much more clearly by using the single-cell data to construct a phase resetting curve (PRC) [32]. The PRC is the deviation of the division time for each event from the unperturbed cell cycle period plotted as a function of the start time of the pulse (relative to the previous division; **Figure 3B**; **Figure S2** in Text S1). In constructing **Figure 3B**, we assume that the responses to successive pulses are independent and pool their phase shifts. This assumption is confirmed by our data (**Figure S3** in Text S1) and is also justified by the fast turnover rate of CtrA [24].

Return map analysis (**Figure S4** in Text S1) reveals that stable phase-locking corresponds to portions of PRCs with slopes between 0 and −2 (see Eqs. (S1)–(S6) and **Figures S5–S8** in Text S1). The experimental PRCs (**Figure 3B**) are further employed in Langevin equation simulations to recapitulate the measured synchronization responses of cell population to external periodic (**Figures S6–S7** in Text S1) and non-periodic (**Figure S8** in Text S1) pulse trains. These results together indicate that the constructed PRCs, which are average responses, are sufficient to capture the division statistics of interest.

### The cell cycle is more readily delayed than advanced

Consistent with the frequency response above, our experimental PRCs demonstrate that the cell cycle response to CtrA pulsation exhibits greater delay than advance. This asymmetry is our main experimental result. Our finding is surprising considering the measured CtrA temporal concentration profile, which has a slow rise and a rapid fall (∼70 min and ∼10 min, respectively, under conditions with a stalked cell period of ∼80 min) [33]. Perturbation pulses that occur during the rise will tend to advance the CtrA oscillation, while pulses applied during the fall will tend to delay the CtrA oscillation (**Figure 3C**). The slow rise and rapid fall should thus favor advance over delay. A mathematical model that is based on current molecular knowledge [19] also exhibits a much more pronounced advance than delay, regardless of the choice of parameters (**Figure S9** in Text S1). The behavior of the molecular model can be understood as follows. CtrA accumulates during the stalked phase and peaks at the pre-divisional stage. This accumulation positively feeds to a proteolytic system that rapidly turns over CtrA within a short time. In this way, the different modules function like gears in a machine—there is no clutch to allow variable coupling between the “engine” and “transmission”, and cell division is locked to CtrA oscillation.

The CtrA-dependent PRCs that we obtained from our measurements are inconsistent with an explicit gear-like mechanism (see **Figure 3D**). Corroborating this idea, the strict concentration dependence of a gear-like mechanism would predict that a decreased amplitude of the regulatory signal should either block or delay cell cycle events [4]. Indeed, in the above mathematical model [19], a reduced *ctrA* induction level leads to a reduced amplitude of its oscillation and a longer period. However, we showed that *C. crescentus* cells yield similar reproduction cycle time distributions for a wide range of constant inducer concentrations [20] (**Figure S1** in Text S1). Furthermore, the fact that the functional modules of the regulatory network need not all move forward at the same pace and can even run independent of the cell cycle [6]–[9] suggests that coupling of multiple (autonomous) oscillators is a fundamental feature of the system.

### Elucidation of the form of the coupling between functional modules

**Figure 4:**
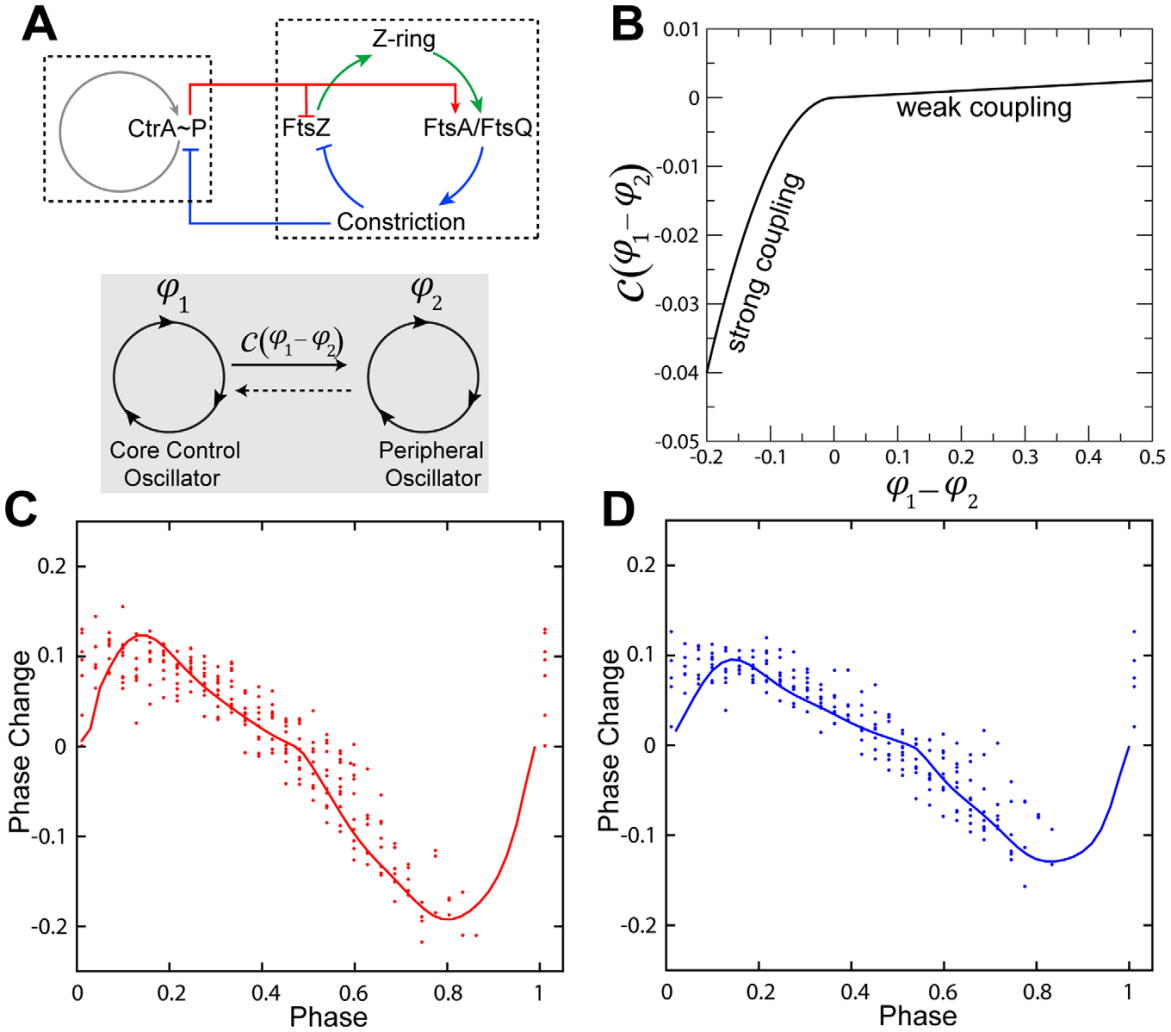
Proposed coupled-oscillator model for *C. crescentus* cell cycle control. (**A**) Interactions between core cell cycle regulatory module and cell division module. Cell division module is represented as looped connections of protein expression and interaction events. This closed loop is established by both protein interaction causalities and temporally connected events. The color scheme is the same as **Figure S9** in Text S1. The interactions are schematized in the lower panel. (**B**) Derived coupling function 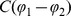. See **Text S1** for details. (**C**) Reverse-calculated PRC overlapped with experimental PRC data for 15-min pulses. (**D**) Comparison between experimental PRC data for 10-min pulses and calculated PRC based on the coupling function in (**B**) derived from 15-min-pulse data. See **Text S1** for details.

Our point is not to argue for or against any particular molecular model but to show that our systems-level measurements are qualitatively inconsistent with extrapolations of behavior from the known molecular interactions. To interpret our data, in particular the PRC, we introduce a simple phenomelogical model that reveals systems-level information and can guide future studies. It comprises a core module (subscript 1 in **Eq. (1)**) that is coupled to a peripheral division module (subscript 2 in **Eq. (1)**) (**Figure 4A**):
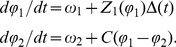
(1)Here, 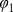 and 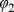 are the phases of the CtrA oscillator and the cell division oscillator respectively, 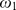 and 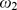 are the corresponding intrinsic phase velocities, 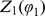 describes the response of the core module to a time-dependent perturbation 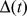, and 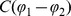 is the coupling from the core CtrA oscillator to the cell division oscillator and is a function of the phase difference. The function 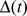 encodes the CtrA pulse train (i.e., it is 1 for the duration of each pulse and 0 otherwise). We take for 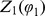 the derivative of the PRC of an existing model of the CtrA oscillator [20] (**Figure S10** in Text S1and Text S1). To elucidate 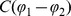 (**Figure 4B**) we begin by noting that the perturbation never results in a stable phase difference other than the original one (see Methods). Consequently, we know that the system has a single stable point, and we can choose the zeros of 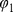 and 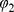 such that it occurs at 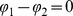. Mathematically, 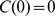 and 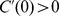, where the prime denotes differentiation. The slope of 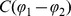 sets the relaxation rate; the relaxation rate in turn sets the extent of the advance when 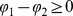 and the extent of the delay when 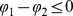. We adjusted the slopes of line segments for 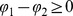 and 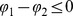 separately to match the advance and delay observed in the 15-min pulse experiment. In this model, the phase advance of the CtrA oscillator is weakly coupled to the division oscillator, while the delay is strongly coupled (**Methods**).

We test the model and the elucidated coupling function by using it, without further modification, to compute the measured PRC obtained with 10-min pulses (**Figure 4C–D**). This is a non-trivial test since the 10-min-pulse PRC is not a simple scalar multiple of the 15-min-pulse PRC. We see that the agreement is excellent. Crucially, the model captures the fact that the asymmetry between delay and advance is less pronounced for 10-min pulses. An additional prediction of this coupled oscillator model is that the cell cycle will become more gear-like with stronger coupling and less gear-like with weaker coupling. Indeed, weakening the coupling by lowering the amplitude of the first oscillator by decreasing the *ctrA* induction level reduces the coherence of the second oscillator output due to the presence of increased noise (i.e., the ratio of standard deviation of the cell inter-division time distribution over mean inter-division time increases, **Figure S1** in Text S1). Meanwhile, multiple cell constrictions that occur within a cell cycle [7]–[9] could be explained by “phase slip” between the autonomous CtrA and division modules. This asymmetrically coupled oscillator picture thus provides a theoretical foundation to explain the experimentally observed bacterial cell-cycle defects.

### Molecular interpretation

What molecular components could make up the autonomous oscillator downstream of the core CtrA module? A self-sustained oscillator requires negative feedback with sufficient time delay [34], [35]. Examination of the molecular details identifies the existence of an appropriate motif in the FtsZ-FtsQA interactions (**Figure S11** in Text S1): i) the residual transcription activity of *ftsQ* and *ftsA* from the P_aq_ promoter (∼25% of normal activity, estimated from [36]) in the absence of CtrA may yield sufficient expression of FtsQ and FtsA; ii) transcription of *ftsA* from the P_a_ promoter is independent of CtrA [36]; iii) the time difference between FtsZ expression and Z-ring formation may provide sufficient time delay for the feedback loop; iv) the cell phase-dependent proteolytic property of FtsZ provides a negative feedback signal, i.e., the half-life of FtsZ decreases rapidly as Z-ring constriction initiates [37]. Thus Z-ring formation contributes to a time-delay while Z-ring constriction negatively regulates the stability of the division proteins. These details are encapsulated in the coupled oscillator scheme of **Figure 4A**.

### Broader implications

The present study is an important step beyond the recent work using simple synthetic biological oscillators [28] because we can exploit the dynamics to learn about the natural organization of the cell cycle and its design principles. Our findings are congruent with the recent observation that DnaA activity, which controls DNA replication, oscillates independently of CtrA [5], and, more generally, the “phase-locking” model proposed by Lu and Cross for budding yeast [4]. In the yeast model, the central cyclin/Cdk oscillator entrains a series of autonomous peripheral oscillators with intermittent coupling. Corroborating this picture, periodic *CLN3* expression indicates that certain checkpoints in the yeast cell cycle can be abolished [38].

Given that the coupled-oscillator topology appears in the cell cycle control of multiple organisms, it is important to consider its implications and functional advantages. While the CtrA module is often viewed as the “engine” of the cell cycle, our results show that it cannot significantly accelerate division; rather, it appears to function more like a brake, slowing downstream events. This could be important for ensuring coordination of the many processes that contribute to the cell cycle. The asymmetric, diode-like, coupling function in **Figure 4B** will also affect the propagation of noise from the upstream module to the downstream one. To show this, we added white noise terms to both oscillators in **Eq. (1)**. The upstream (i.e., 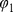) noise propagates to the downstream (observed) phase through asymmetric coupling, giving rise to a skewed distribution in the (unperturbed) division times (**Figure S12** in Text S1). To reproduce the experimental distribution, the upstream noise needs to be ∼10-fold greater than the downstream noise (**Figure S12** in Text S1and Text S1). Thus, the coupled-oscillator topology filters perturbations/fluctuations that advance the CtrA oscillator phase. This reduction in noise, in turn, would prevent premature division. In addition, it would be also interesting to investigate the robustness and stability of this coupled-oscillator model through systematic non-equilibrium theoretical frameworks [39]–[41].

The approach that is presented here builds on the basic principle of linear response, which is central to spectroscopy and engineering. In this sense, it is a chemical perturbation spectroscopy (CPS) [42], [43]. The parameterless fit of the 10-min-pulse PRC data with the model determined from the 15-min-pulse PRC establishes its suitability in the present case. Our work transcends recent linear response studies of cellular networks [44]–[48] by going beyond the steady-state to determine the full cell cycle response to pulsatile perturbation, as represented by the PRC. In this sense, it is most similar to [49] but we focus on extracting topological features of the regulatory dynamics rather than discriminating between specific molecular models. This analysis can be adapted to study oscillatory dynamics in other cellular systems. In the future, we envision multiple chemical perturbations, potentially with more complex waveforms, that could directly probe the bidirectional information flux between functional modules, in analogy to multi-dimensional (NMR and optical) spectroscopies [50].

## Methods

### Construction and characterization of *C. crescentus ctrA* mutant strains

FC1006 was constructed by substituting the defective holdfast synthesis gene *hfsA* [51] in LS2535 (NA1000 Δ*ctrA*+P*xylX::ctrA*) with CB15 *hfsA* allele [26] by double-recombination. The CB15 *hfsA* allele-containing plasmid was introduced to LS2535 by tri-parental mating from Top10/pNPTS 138-CB15-*hfsA* [26] and was selected on a 20 µg/ml kanamycin PYE plate supplemented with 0.3% xylose. Colonies were grown overnight without kanamycin selection to allow recombination, counter-selected on a sucrose containing plate, and then tested on a kanamycin plate to ensure the loss of kanamycin resistance. The successful recombinant was screened by the adhesion phenotype with the 96-well crystal violet assay [26], and confirmed by PCR amplification (MEN-SNP-70 primers, TCCCGGTCCAGTTTCAGC and AAGTACGCGGTGGCTTCG) and restriction enzyme digestion with AvaI and BstNI. The resulting FC1006 strain has ∼30% of the surface adhesion ability of wild-type CB15 after 5 hrs of induction with ≥0.03% xylose as characterized by the polystyrene binding assay. The FC1071 strain was constructed by introducing the P*xylX::ctrA* plasmid [24] from LS2535 into FC764 (NA1000 with CB15 *hfsA* allele [26]).

### Cell culture

Individual colonies (FC1006 or FC1071) were picked from a fresh PYE agar plate supplemented with necessary antibiotics and xylose (1 µg/ml chloramphenicol, 0.03% xylose) and grown overnight in PYE medium (1 µg/ml chloramphenicol, 0.03% xylose) in a 30°C rolled incubator. The overnight culture was diluted to OD_660_ = 0.1 with fresh PYE (with antibiotics and xylose) and cultured for additional 2 hrs before loading into the microfluidic device with a syringe [20].

### Microfluidic device and single-cell assay

Y-shaped microfluidic channels with rectangular cross-section (150 µm width×50 µm height) were fabricated by rapid phototyping in poly(dimethylsiloxane) (PDMS) [52]. The PDMS and a microscope coverslip (No. 1.5) were plasma cleaned and then pressed and sealed to form Y channels with inlet and outlet ports in the PDMS. Each device contains multiple channels allowing simultaneous measurements under different conditions. Teflon tubing connectors (constructed with i.d. 0.028″/o.d. 0.048″ tubing and i.d. 0.045″/o.d. 0.062″ tubing) plugged with i.d. 0.012″/o.d. 0.030″ tubing were connected to ports and used for solution exchange. Before loading the bacterial cell culture, the channel was sequentially rinsed with NaOH (2M), ethanol, and autoclaved H_2_O. After thermal equilibration inside the heated microscope enclosure and incubator, the channel was loaded with the bacterial cell culture. Generally, ∼1 hr incubation for FC1006 or ∼30 min incubation for FC1071 is necessary for a sufficient number of single cells to become attached onto the glass surface of the channel. Two computer-controlled syringe pumps (PHD2000, Harvard Apparatus) that are also inside the heated (thermostated) microscope incubator were used to pump two thermally equilibrated PYE media with low and high xylose concentrations through the channel at a constant flow rate (10 µL/min) [20].

### Time-lapse microscopy

Time-lapse single-cell measurements were performed on an automated inverted microscope (Olympus X70) equipped with a motorized sample stage, an objective motor driver and a controller (BioPrecision stage and MAC5000 controller, Ludl Electronics). DIC microscopy was done with an Olympus UPLSAPO 100× oil objective and a light-emitting diode (LED) light source which is pulse-modulated (LEDC19 LED and LEDD1 driver, Thorlabs). The control pulse for the LED was generated from a PCI-DAQ card (PCI-6052E, National Instrument) through a BNC adaptor interface (BNC-2090, National Instrument). The image was collected on a charge-coupled digital camera (CCD, LCL-902C, Watec) with total magnification of 100×. To ensure thermal stability, most of the microscope (except for the observation ports) as well as the syringe pumps were enclosed by a home-made acrylic microscope enclosure (28″×25″×18″) heated with a heater fan (HGL419, Omega), and the temperature was maintained at 32°C by a proportional integral derivative temperature controller (the “incubator” mentioned above; CSC32J, Omega). A uniform temperature profile inside the incubator is achieved by active air flow from two small-profile heaters inside the enclosure.

DIC images of multiple fields-of-view were recorded at 2 frames/min and the focus was adjusted automatically by a total-internal-reflection (TIR) based autofocusing control loop. The back-reflected beam of a TIR-aligned 633 nm laser (LHRP-0081, Research Electro-Optics) impinges on a quadrant photodiode detector. The amplified difference signal is the error signal that is used as a feedback for adjustment of the objective (motor) position. A Virtual Instrument routine (LabView 7.0, National Instrument) was used to control all the components (i.e., sample stage, autofocus, pumps, CCD, and LED) and run the experiment for extended (>20 hrs) periods of time.

### Data analysis and construction of population growth curves

The stack of acquired DIC images was loaded into ImageJ (NIH) and the division events of individual cells were tracked manually and recorded by a home-made plug-in. Cells that grew into long filaments or stopped reproduction were excluded from the analysis. The division event data was imported into Matlab (MathWorks) and processed. A typical periodic perturbation data set contained >200 cells. Note that only the original set of stalked cells was used for the present analysis; we did not include cell division data from any of the progeny cells that adhered to the glass surface.

Growth curves were constructed from the division event data for individual cells. Each observed division contributes to a unit increase in the population size. Since we only followed continuous reproduction of the initial population of stalked cells on the glass surface, the total number of cells generated by this population of cells can be represented by the differential equation 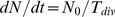, where 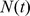 is the number of total number of cells as a function of time, 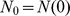, and 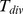 is the division time of cells. Therefore, linear growth behavior is expected for the asynchronous division of a population of cells.

### Construction of phase difference distribution and characterization of synchrony

The phase difference is defined as the temporal difference between the time of each cell division event (i.e., each dot in **Figure 2B**) and the start time of the subsequent high inducer pulse (depicted in **Figure S3a** inset in Text S1). Without dispersion in the population, a single phase difference is present for a phase locking condition [32]. However, the existence of noise leads to a distribution of phases for the population under phase locking. To construct this distribution, we included division events between the start times of two successive high inducer pulses for periodic perturbations with an external period longer than 65 min; and for external periods shorter or equal to 65 min, we included division events between the end times of two successive high inducer pulses.

The synchronization index [31] is calculated based on the phase difference distribution, which is based on the Shannon Entropy: 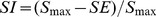, where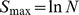, 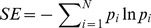, 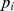 is the probability at each state and *N* is the number of bins. *SI* ranges from 0 (uniform phase distribution) to 1 (singular phase occupation). In all of our calculation, we use a constant bin number of *N = 20*. The evolution of phase locking is characterized by the arithmetic mean of the phase difference distribution at different multiples of the external period (**Figure 2** and **Figure S5** in Text S1).

### Construction of experimental phase response curves from cell division times

With the assumption that phase responses of individual cell cycle oscillators are independent of the pulse number (which is validated in the **Text S1**), we constructed the phase response curve from the data of all pulses in each constant pulse period experiment. The scattered data (**Figure S3B** in Text S1) within a chosen bin range (i.e., 2 min as represented by the gray bar) were used to construct perturbed cell cycle time distributions (insets). For the distributions which are obviously truncated due to data sampling limitations, we used the center of the fitted Gaussian as the perturbed cell cycle time; while for other distributions, the arithmetic mean values are used instead as the reset cell cycle time.

By this approach, we obtained a set of phase response data (i.e. phase vs. perturbed cell cycle time) for each *t_H_*. The results for *t_H_* = 10 min and 15 min are shown in **Figure 3B** **and Figure S2** in Text S1, where the perturbed cell cycle time is converted to phase advance or delay and the phase difference in minutes is scaled to be between 0 to 1 by the mean native cell cycle time at low inducer concentration (68.1 min). The missing data points for phase approaching unity are due to the finite width of high inducer concentration pulse. The phase response data at the minimum phase (i.e., phase = 0.011) are duplicated to indicate the periodic nature of phase response curves (i.e., these data are duplicated at phase = 1.011). These data are then fit with a trigonometric polynomial of degree three to ensure periodicity: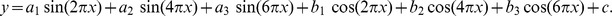 The fit parameters (a1, a2, a3, b1, b2, b3, c) for phase response curves at *t_H_* = 10 and 15 min are (5.58725, 1.05375, −0.03266, 1.585, 1.29886, 0.69753, 0.72275) and (8.41916, 1.60012, −0.32122, 1.7194, 2.57242, 1.06958, −0.87352), respectively.

### Assumptions underlying determination of the coupling function

We derive the form of Eq. (1) in **Text S1**, starting from a classic mathematical description of interacting oscillators [53]. We estimate the sensitivity function 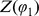 from the gene regulatory network of the CtrA module [20]; more precisely, in numerical practice, we approximate 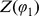 as constant over the duration of the pulse, with its value given by the published function at the phase when the pulse begins. The specific choice of the model in [20] does not significantly affect the result. In determining the coupling function 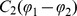 as described in the Results and Discussion, we first analyze the steady-state solution in the case when there is a single pulse by assuming that: (1) 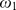 and 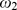 are equal to each other and (2) the effect of a pulse on the first oscillator is equally distributed throughout its duration. If the response of the first oscillator to the pulse is small and the steady-state is stable, the second oscillator will maintain the initial phase difference 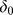. If the response of the first oscillator to the pulse is large, the second oscillator can be displaced to a new stable solution 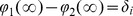 for 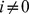. The criterion for a solution to be stable is 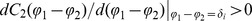. Because we do not observe discontinuous responses in the experiments, we conclude that the response of the first oscillator is not strong enough to allow solutions other than 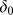. Rather than being instantaneous, the time required for the second oscillator to relax back to the initial state 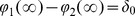 is set by the slope, with steeper slopes leading to faster relaxation.

## Supporting Information

Text S1 Supporting information including supplementary text and figs.
(DOC)
