# Supplementary material for: Phase Resetting Reveals Network Dynamics Underlying a Bacterial Cell Cycle
Source: PLoS Comput Biol. 2012 Nov 29;8(11):e1002778. doi: 10.1371/journal.pcbi.1002778 (PMC3510036; doi:10.1371/journal.pcbi.1002778)
Supplement: Text S1 — Supporting information including supplementary text and figs. (DOC) [file pcbi.1002778.s001.doc]

**Phase resetting reveals network dynamics underlying a bacterial cell cycle**

Yihan Lin^1, 4^, Ying Li^2, 4^, Sean Crosson^3^, Aaron R. Dinner^1, 4, *^, and Norbert F. Scherer^1, 4, *^

Department of Chemistry^1^, Physics^2^, and Biochemistry and Molecular Biology^3^, and Institute for Biophysical Dynamics^4^, University of Chicago, 929 East 57^th^ Street, Chicago, IL 60637

*E-mail: [dinner@uchicago.edu](mailto:dinner@uchicago.edu); [nfschere@uchicago.edu](mailto:nfschere@uchicago.edu)

**Text S1**

**Contents**

1. Assumption of fast relaxation of cell cycle oscillator after perturbation
2. From PRC to phase-locking: single-oscillator description and Poincaré return map analysis
3. Numerical simulation of responses of noisy single phase oscillators to periodic and non-periodic perturbation
4. Comparison between experimental and simulated PRCs
5. Derivation of coupled-oscillator model
6. **Assumption of fast relaxation of cell cycle oscillator after perturbation**

Conventional oscillator perturbation experiments are based on isolated perturbations [1]. The phase response from such experiments is constructed by measuring the phase change stimulated by a single pulsed perturbation after sufficient relaxation of the oscillator back to its free-running mode (a limit cycle). In contrast, our analysis of phase response is based on periodic perturbation and assumes relatively fast relaxation to the steady-state after perturbation. To validate this assumption, we constructed the phase response curves from single cell measurements for each (different) external pulse period in a single periodic perturbation experiment (*t_H_* = 15 min and *T* = 80 min) (**Figure S3a**). Qualitatively, the data from the different periods of the chemical perturbation lie within the same cell cycle dispersion envelope (i.e. along the Y-axis) and do not differ from each other in terms of dispersion. Moreover, the data from different low inducer durations (i.e., different relaxation times after perturbation) overlap well (**Figure S3b**) except for the region where the sampling range of the cell cycle time is truncated due to construction (as shown in the insets). Therefore, it is valid to assume that the phase response curve obtained from our periodic perturbation experiments agrees with that from single pulse perturbation experiments.

1. **From PRC to phase-locking: single-oscillator description and Poincaré return map analysis**

By definition, a stable (amplitude) oscillator returns to its limit-cycle attractor after exposure to small amplitude perturbations. Regardless of the detailed molecular mechanism, the dynamics of the cell cycle oscillator in the presence of zero or small amplitude perturbations can be represented by its phase variable [2],

(S1)

where is the intrinsic phase velocity,is the sensitivity function,$\varepsilon(t)$ is a small external force that is applied as a function of time, and $F(t)$is the noise. Integration of over $\Delta t$, the duration of a single square wave perturbation, gives the phase resetting curve [2]:

. (S2)

For a periodic train of square pulses with frequency $f_{ext}$, which interacts with and entrains the cell cycle (i.e., the internal) oscillator about its fundamental frequency (termed 1:1 mode), we can characterize the phase-locking region and stability with Poincaré return map analysis [3]. The phase of the oscillator in the *n*th external period, , and its phase after another external period, , need to satisfy:

, (S3)

where is the intrinsic phase velocity, is the external oscillator period, and . Under phase-locking conditions, and we have

(S4)

or , (S5)

which defines the range of external oscillator frequency that permit phase-locking. The range of internal oscillator phase values that permit phase-locking can in turn be obtained from the analysis of the Poincaré return map of **Eqn. (S5)**. Two fixed points are found (**Figure S4**) and their stabilities can be determined by the slope of the PRC [2,3]. Specifically, for the fixed point to be attractive and stable, it requires:

,

or: . (S6)

Therefore, stable phase locking can be achieved only within the region where the slope of the PRC is between -2 and 0 [3].

An independent approach to identifying the stable phase-locking region is to construct the relation between the stable phase difference and the external period according to **Eqn. (S5)** by using the identified stable phase difference measured for various phase-locking experiments as in **Figure S5a**. Specifically, the stable phase difference in time is converted to phase (between 0 and 1) as the horizontal axis. The difference between external period and internal unperturbed period (68.1 min) is converted as phase change (i.e., advance or delay) for the vertical axis. The constructed relations are overlaid on the PRCs as shown in **Figure S5b** (open circles).

1. **Numerical simulation of responses of noisy single phase oscillators to periodic and non-periodic perturbation**

To examine whether a single oscillator model with Gaussian noise can capture the experimental results, we simulated the trajectory of a single phase oscillator using Eqn. (S1) assuming Gaussian white noise in the simulation. We assumed that the phase in the sensitivity function increases linearly with respect to time during the high inducer pulse, hence Eqn. (S2) becomes:

, (S7)

where is the high inducer pulse duration and is the mean native cell cycle time. With such simplification, we were able to approximate the analytical form of. The purpose is to perform direct simulation of the differential equation **Eqn. (S1)**.

The simulation of Eqn. (S1) was carried out using the method described in [4]. Briefly, a small increment of is calculated by

, (S8)

where is a very small time interval (0.01 min), is the intrinsic phase velocity ($\omega_{0}^{-1}$ = 68.1 min), is a periodic or non-periodic function ($\varepsilon(t)=$1 during high inducer pulse and 0 during low inducer pulse), is the noise amplitude approximated from experimental distribution (0.018), and is a Gaussian random number of zero mean and variance equals to 1. As above, is the sensitivity function; it is important to note that, in applying Eqn. (S8), we assume that is constant over the entire pulse duration, with the value specified by the phase at the time of initiation of the pulse.

A representative simulation with a population of phase oscillators starting with randomly distributed phase is shown in **Figure S6**, which captures the phase locking feature of its experimental counterpart (**Figure 2A**). Simulations conducted for different pulse profiles were used to quantify the synchronization index and phase difference, as have been characterized for the experiments. The trends of both quantities (**Figure S7a and Figure S7 b-c**) agree with the experimental results (**Figure 3a** and **Figure S5 b-c**). Furthermore, simulations with non-periodic external pulse perturbations also capture the behaviors of this cell cycle oscillator measured under non-periodic perturbation experiment (**Figure S8**). It is noted that these simulations only complement the analysis done in the previous section and do not offer new insights.

1. **Comparison between experimental and simulated PRCs**

A key test was to establish whether the published (molecularly detailed) model of the network could capture the experimental PRC. Using a model of the Caulobacter cell cycle from Li and Tyson [5], we simulated the CtrA perturbation experiment and used the response of the model to construct the PRC. This model incorporates details pertinent to the regulation of the cell cycle including known regulatory proteins oscillations and subcellular processes such as DNA replication and Z-ring formation. This model captures the phenotypes of various existing mutants and provides predictions for novel mutants. The *ΔctrA* / *P_xyl_-ctrA* mutant is relevant to our experiment. We adopt most of the model parameters and simulate a single-pulse perturbation experiment to calculate the PRC (**Figure S9a**). Since the unperturbed cell cycle time in the simulation of this mutant is longer than our experimental value (97.1 min vs. 68.1 min), the duration of the simulated pulsed perturbation with elevated *ctrA* induction is scaled accordingly (i.e. 14.3 min and 24.1 min in simulation vs. 10 min and 15 min in experiment). The perturbation amplitude (i.e., the elevated *ctrA* induction rate) was adjusted such that the relaxation from the perturbation is essentially complete within one cell cycle and the magnitude of the phase advance in the simulated PRCs is close to that observed experimentally.

The simulated PRCs exhibit a sinusoidal shape that is similar to the experimental ones. However, the experimental PRCs exhibit much larger phase delay responses when perturbation is administrated during the latter portion of the cell cycle (**Figure S9a**). Since the phase response characterizes the response of the downstream cell division machinery to the perturbation of CtrA concentration, we need to examine by simulation how the perturbation of CtrA concentration propagates through the entire network to the regulation of cell division machinery. Thus, we summarize the major effects of CtrA on various cell division proteins and relevant division processes, including the production of FtsZ, the formation of Z-ring by FtsZ, the production of FtsQ, and the constriction of Z-ring by FtsQ (**Figure S9b**). In the unperturbed cell cycle, the division process starts with the expression of FtsZ in the early stalked cell [6]. The increase in FtsZ leads to formation of Z-ring in the mid-cell plane. As CtrA~P increases rapidly in the late stalked stage, the expression of FtsZ is suppressed while that of FtsQ (and FtsA) is activated [6,7]. The fully-assembled Z-ring begins to be constricted by FtsQ (and FtsA). As constriction progresses, the stabilities of FtsZ [6,8] and FtsQ drop. Turnover of FtsZ and FtsQ accompany the completion of constriction (i.e., cell division). The simulated protein trajectories (solid) in **Figure S9b** reflect the aforementioned regulations. For *t_H_* = 24.1 min simulation, when perturbation on CtrA expression is introduced early in the cell cycle phase (i.e., phase = 0.2), cell division is advanced significantly with a comparable magnitude as the advance in CtrA~P oscillation: the FtsQ peak appears earlier and constriction of Z-ring completes faster, and the magnitude of advance of FtsQ or Z-ring trajectory is comparable to that of CtrA~P (left, **Figure S9b**). However, the delay response of cell division to CtrA perturbation later in the cell cycle (i.e., phase = 0.75) is much less significant (right, **Figure S9b**) than previous situation (left, **Figure S9b**). These molecular-level details of perturbation-response from simulations underlie the shape of the simulated PRC (right**, Figure S9a**). The regulation of CtrA on the division process in the model simulation, i.e., the advance/delay in CtrA oscillation, propagates almost linearly to the advance/delay in the division process.

With these mechanistic insights, we now try to resolve the discrepancy between experimental and simulated PRCs by parameter tuning and limit our focus on the longer *t_H_* duration PRCs (right**, Figure S9a**). Since the perturbation of CtrA is constant, it should be the interaction between CtrA and the cell division process that contributes to the observed discrepancy. And since much of the discrepancy lies in the delayed portion of the PRCs, we need to tune the relevant parameters that would cause larger delay in the cell division with the same CtrA perturbation. Of the options, (i) strengthening the repression of FtsZ by CtrA, (ii) weakening the promotion of FtsQ by CtrA, and (iii) weakening the promotion of Z-ring constriction by FtsQ, only the first option can be realized in the model. Tuning (ii) and (iii) lead to instability in the simulation (left, **Figure S9c**). However, even a ~30% decrease of the CtrA-FtsZ repression constant leaves the PRC almost unchanged (right, **Figure S9c**). Thus, this inability to achieve the measured PRC by adjusting parameters of the model suggested that the discrepancy was more fundamental in nature.

1. **Derivation of coupled-oscillator model**

As discussed in the main text, there is evidence that cell cycle modules oscillate autonomously and we interpret measurements with a coupled oscillator model. Based on classic mathematical descriptions of interacting oscillators [9], we start from the ordinary differential equations for the concentration of proteins ($\vec{X}_{1}$and $\vec{X}_{2}$) in these two modules:

$\begin{matrix} \frac{d\vec{X}_{1}}{dt}=\vec{F}_{1}(\vec{X}_{1})+\varepsilon\vec{V}_{1}(\vec{X}_{1},\vec{X}_{2}) \\ \frac{d\vec{X}_{2}}{dt}=\vec{F}_{2}(\vec{X}_{2})+\varepsilon\vec{V}_{2}(\vec{X}_{1},\vec{X}_{2}), \end{matrix}$ (S9)

where vectors $\varepsilon\vec{V}_{1}(\vec{X}_{1},\vec{X}_{2})$ and $\varepsilon\vec{V}_{2}(\vec{X}_{1},\vec{X}_{2})$ weakly couple the two oscillators ($\varepsilon$ is small). In addition, we assume that the frequencies of the two oscillators are close to each other when there is no coupling. To emphasize this feature, we rewrite $\vec{F}_{i}$ as

$\vec{F}_{i}(\vec{X}_{i})=\vec{F}(\vec{X}_{i})+\varepsilon\vec{f}_{i}(\vec{X}_{i})$. (S10)

To apply this formalism to the present case, we need to transform the concentration representation into a phase representation. We denote the phases of the two oscillators as and . We choose the units of time such that . Comparison with **Eqn. (S9)** with $\varepsilon=0$e = 0 and the chain rule then suggest

(S11)

Below, we denote as to reflect its role as the phase response function. In this way, the equations of motion of the phases are

, (S12)

where and .

As an approximation, we consider the influence of the coupling terms on the phases to be homogenous for all values and integrate over the period $T$ and (small$\varepsilon$). **Eqn. (S12)** becomes:

(S13)

where and .

Now, we consider the situation in which the *ctrA* induction rate follows a periodic square wave $\vec{\Delta}(t)$:

$\begin{matrix} \frac{d\vec{X}_{1}}{dt}=\vec{F}_{1}(\vec{X}_{1})+\varepsilon\vec{V}_{1}(\vec{X}_{1},\vec{X}_{2})+\vec{\Delta}(t) \\ \frac{d\vec{X}_{2}}{dt}=\vec{F}_{2}(\vec{X}_{2})+\varepsilon\vec{V}_{2}(\vec{X}_{1},\vec{X}_{2}). \end{matrix}$. (S14)

Following the same transformation, the phase equations are

(S15)

$\text{where }\omega_{i}$ are the same as above.

In the experiments, the readout is the phase response curve of the cell division oscillator in the presence of periodic *ctrA* induction. We ignore in the equation for the CtrA oscillator because we assume that the external driving force $\vec{\Delta}(t)$ is larger than the coupling $\varepsilon$. This is reasonable because the effect of the external driving on the second oscillator is of order $\varepsilon\Delta$ but the effect on the first oscillator is of order $\varepsilon^{2}\Delta$ because the perturbation must feed back from the second oscillator. These considerations yield Eqn. (1) in the main text.

**Supplementary Figure Captions**

**Figure S1. Cell cycle time distributions for single cells of *ctrA* mutants for constant xylose conditions.** Division times of FC1006 are characterized for different constant xylose concentrations. Specifically, the statistics are 68.1 ± 15.6 min for 0.00009% xylose (mean ± SD, N=5160), 64.2 ± 9.0 min for 0.00027% xylose (N=444), and 65.5 ± 9.2 min under 0.03% xylose (N=244). And for reference, 65.3 ± 9.9 min for FC1071 for no xylose (N=311). The numbers of “superfast” division events (i.e. below or equal to 33min) are 14 out of 5160 for the 0.00009% condition, 2 out of 444 for the 0.00027% condition, and zeros for the other two.

**Figure S2. Phase resetting curve for *t_H_* =10min.** The data (open circles) are fitted with real trigonometric polynomial of degree three (solid line) to ensure periodicity.

**Figure S3. Fast relaxation of this cell cycle oscillator after perturbation. (a)** Phase responses from different external pulse periods from a single periodic chemical perturbation experiment. The data are from the same periodic perturbation experiment (*t_H_* = 15min and *T* = 80min). The responses (characterized as perturbed cell cycle time) for different perturbations are separated and plotted. The inset illustrates the construction of the x and y components in the plot. **(b)** Phase responses for different low inducer durations (i.e. relaxation time) at *t_H_* = 15min: *T* = 80min and *T* = 59min. The responses from all pulse periods for each periodic condition are plotted. The inset in the upper left corner shows the distribution of perturbed cell cycle time for the 8 ≤ phase <10 min at *T* = 59min and the inset in the lower right corner shows the distribution for 54 ≤ phase <56 min at *T* = 80min. Both distributions are fitted with a Gaussian (red).

**Figure S4. Poincaré return map analysis and experimental construction of stable phase-locking region.** Poincaré return map for **Eqn. (S4)**. Dashed line represents. Solid curve maps the initial phase onto itself by using PRC at *t_H_* =15min at a certain *T_ext_* value. It intersects with diagonal line giving rise to a stable fixed point (solid) and an unstable fixed point (open).

**Figure S5. Phase differences between internal cell cycle oscillator and external oscillator at phase locking agree with phase response curves. (a)** Phase evolution under various external oscillator profiles. The upper panel includes data for 10 min high xylose concentration pulse duration (*t_H_* = 10 min) with external oscillator period ranging from 59 to 86 min. The bottom panel includes data for *t_H_* = 15 min with external oscillator period ranging from 56 to 89 min.

**(b)** Phase difference between internal cell cycle oscillator and external oscillator at phase locking for a variety of external periods with *t_H_* =10min. The phase differences from 8^th^ pulse to 12^th^ pulse for the experimental conditions shown in **(a)** are overlapped with phase response curve (solid) from **Figure 3B and Figure S2**. **(c)** Analogous plot as (a) for *t_H_* =15min.

**Figure S6. Phase locking simulations with Eqn. (S1).** Numerical simulation counterpart for experiment shown in **Figure 2A**.

**Figure S7. Simulated phase locking dynamics capture experimental measurements. (a)** Counterpart of **Figure 3A** from simulation. **(b-c)** Counterparts of **Figure S5 b-c**.

**Figure S8. Synchronization of single *C. crescentus* cells by non-periodic external perturbations with *t_H_* = 15min. (a)** Experiment trajectories measured for different non-periodic perturbation profiles. Top panel shows the perturbation with periods (i.e. *T*) as Gaussian random numbers with mean of 75 min and variance of 25 min^2^. Middle panel shows the perturbation with a frequency “up-chirp”, i.e. the period decreases by 2min in each successive interval. Bottom panel shows the perturbation with a frequency “down-chirp”, i.e. the period increases by 2min successively. **(b)** Simulation counterparts for the experiments in (a).

**Figure S9.** **Discrepancies between our experimental PRCs and simulated PRCs from the Li-Tyson model [5].** **(a)** Comparison between experimental and simulated PRCs. The simulations are carried out based on the *ΔctrA* + constitutive *P_xyl_-ctrA* mutant as described (Figure 8 of [5]) with *k’_ctrA_* modified to 0.03. This mutant has a native division time of 97.1min without perturbation and a finite width square-wave perturbation (*k’_ctrA_*: 0.03 🡪 0.05) is introduced at various cell cycle phases. The cell cycle phase change is quantified by the change in cell cycle time. Left: Perturbation simulation is done with *t*_H_ = 14.3min to compare with *t*_H_ = 10.0min experiment (i.e. *t*_H_ occupies the same fraction of cell cycle time). Right: Perturbation simulation is done with *t*_H_ = 21.4min to compare with *t*_H_ = 15.0min experiment. **(b)** Molecular details of the perturbation-response simulations. (Top) Regulatory circuit for Z-Ring formation and constriction. Transcription of *ftsZ* starts early in the stalked cell cycle and the level of FtsZ protein, which assembles into the Z-Ring at the mid-cell plane, peaks at the late stalked stage while its transcription is repressed by CtrA~P. The regulators for Z-Ring constriction, FtsQ and FtsA, are transcribed from a common promoter *P_QA_* which is induced by CtrA~P at the late stalked stage. (Left) The perturbed and unperturbed trajectories of various protein species in the Li-Tyson model [5] that are subject to square-wave *ctrA* induction perturbation in the early cell cycle phase. The perturbation time window is indicated by the filled box. Unperturbed trajectories are shown as solid curves while the perturbed ones are in dashed. (Right) Same as (Left) for perturbation introduced at a later cell cycle phase. **(c)** Tuning of the network parameters fails to capture experimental PRC. (Left) Permissive and restrictive nodes for parameter tuning. The green box indicates the interaction (CtrAP ―| FtsZ) whose binding parameter (i.e. JiFtsZCtrA [5]) can be changed by >10%. The pink boxes indicate the opposite cases (JiFtsQCtrA for CtrAP ―> FtsAQ and JZFtsQ for FtsAQ ―> Constriction). (Right) The PRC from model simulation is not sensitive to the allowed range of parameter changes. The PRC obtained by lowering JiFtsZCtrA by ~30% (i.e. 0.7 🡪 0.5, maximum allowed change) is shown (blue) and compared with the PRC without parameter change (green, same as the one in the right panel of **(a)**).

**Figure S10.** **Phase resetting curves of the core regulatory module (CtrA) based on our previous model** [10]. (Left) *t_H_* = 10 min. (Right) *t_H_* = 15 min.

**Figure S11.** **Schematic of transcription regulations and protein interactions for Z-ring assembly and constriction.** CtrA~P interacts with division process by transcription regulation of *ftsZ* and *ftsQA*.

**Figure S12.** **Asymmetric coupling produces skewed cell cycle noise as observed in experiment.** Eqn. (1) in the main text was simulated with noise to produce the cell cycle time distribution under unperturbed condition and to compare with experiment (blue here; gray in **Figure S1**,). White noises ($\xi_{1}$,$\xi_{2}$ ) are attributed to the two oscillators, and respectively. The simulated distribution (green) is generated with$\left\langle\xi_{1}^{2}\right\rangle=0.001$and $\left\langle\xi_{2}^{2}\right\rangle=0.0001$.

**Supplementary Figures**

**Figure S1.**


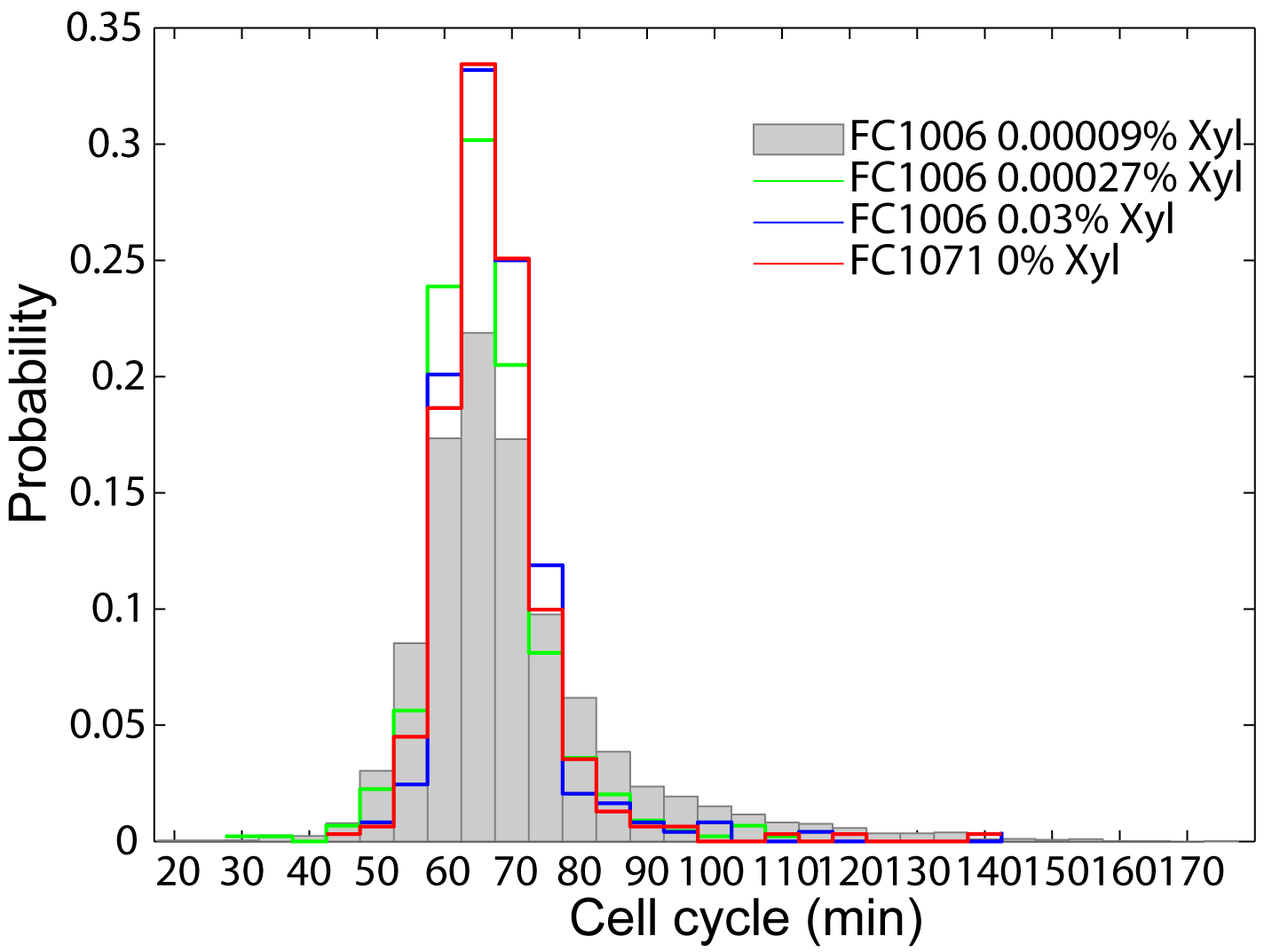


**Figure S2.**

**
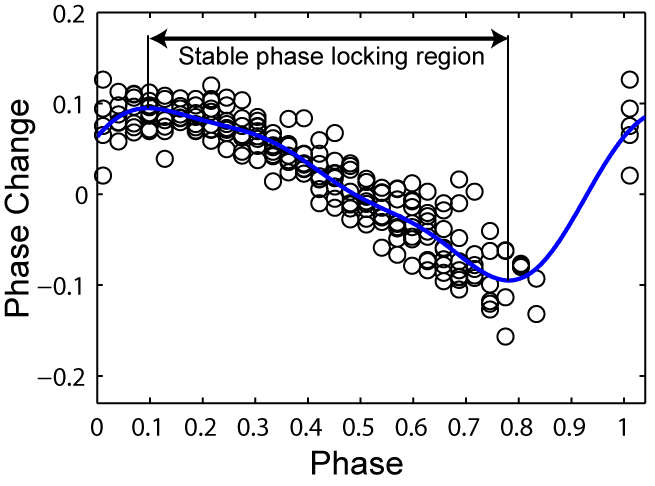
**

**Figure S3.**


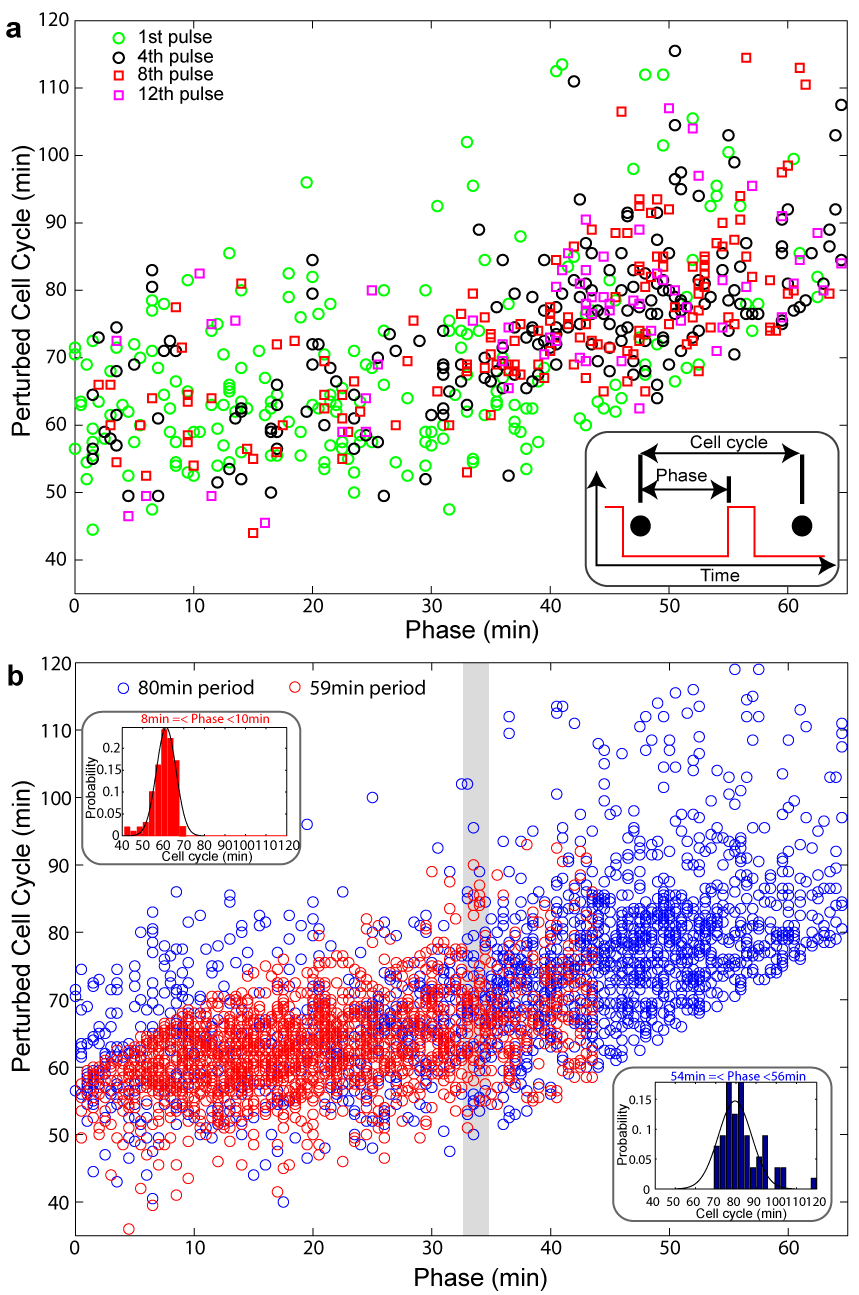


**Figure S4.**

**
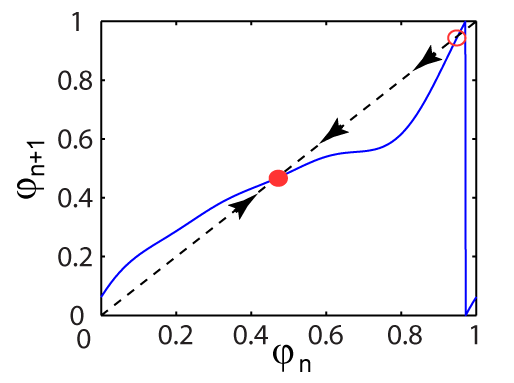
**

**Figure S5.**


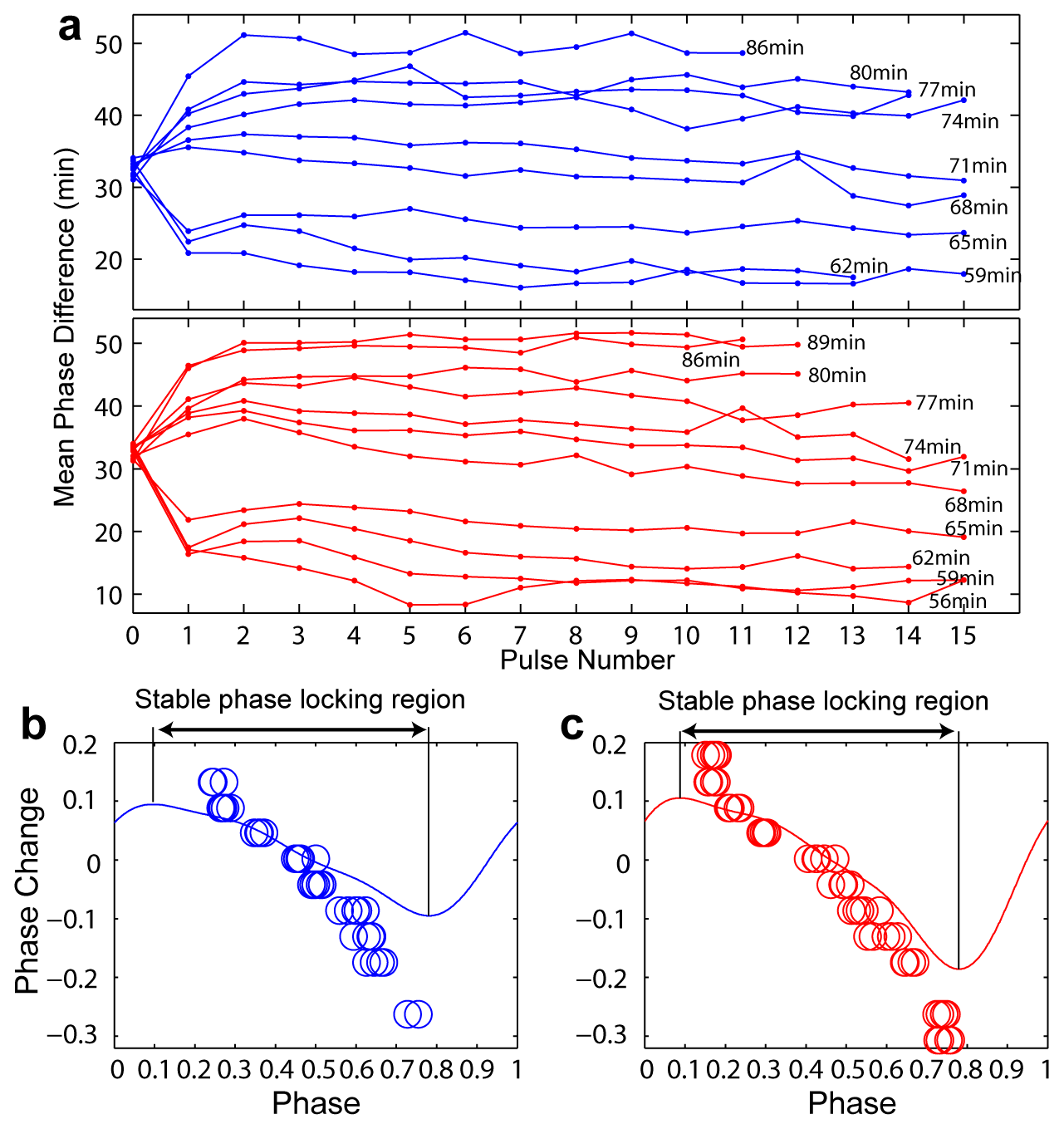


**Figure S6**

**
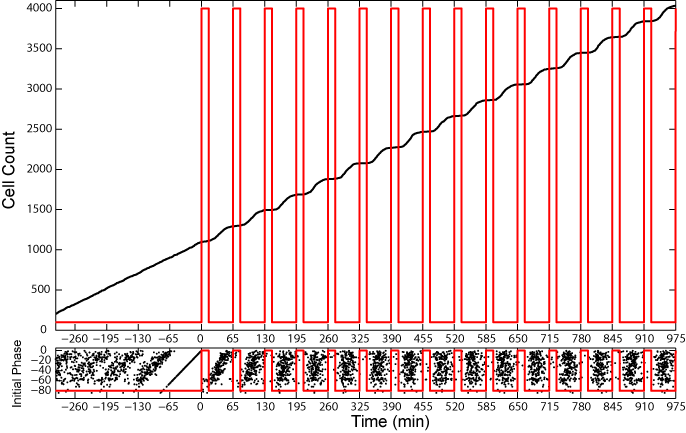
**

**Figure S7.**


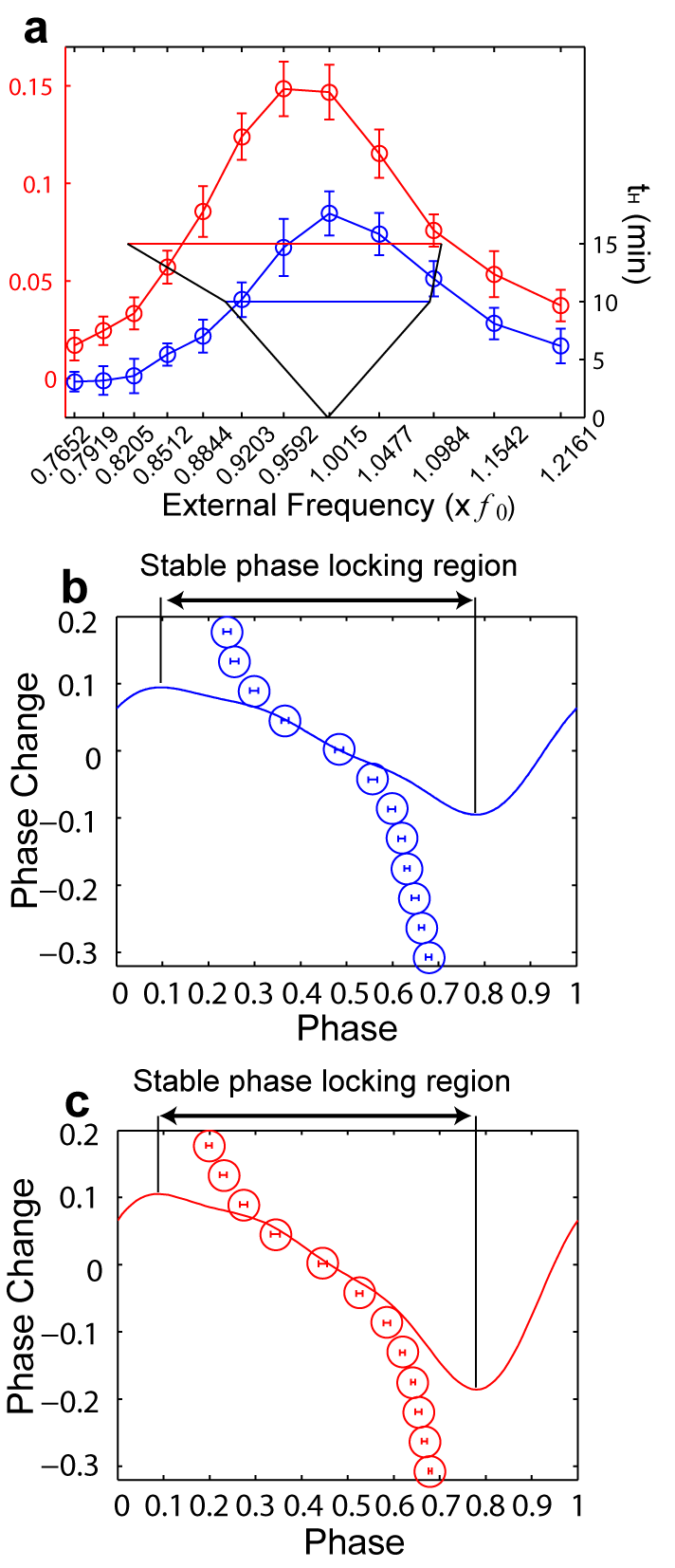


**Figure S8.**

**
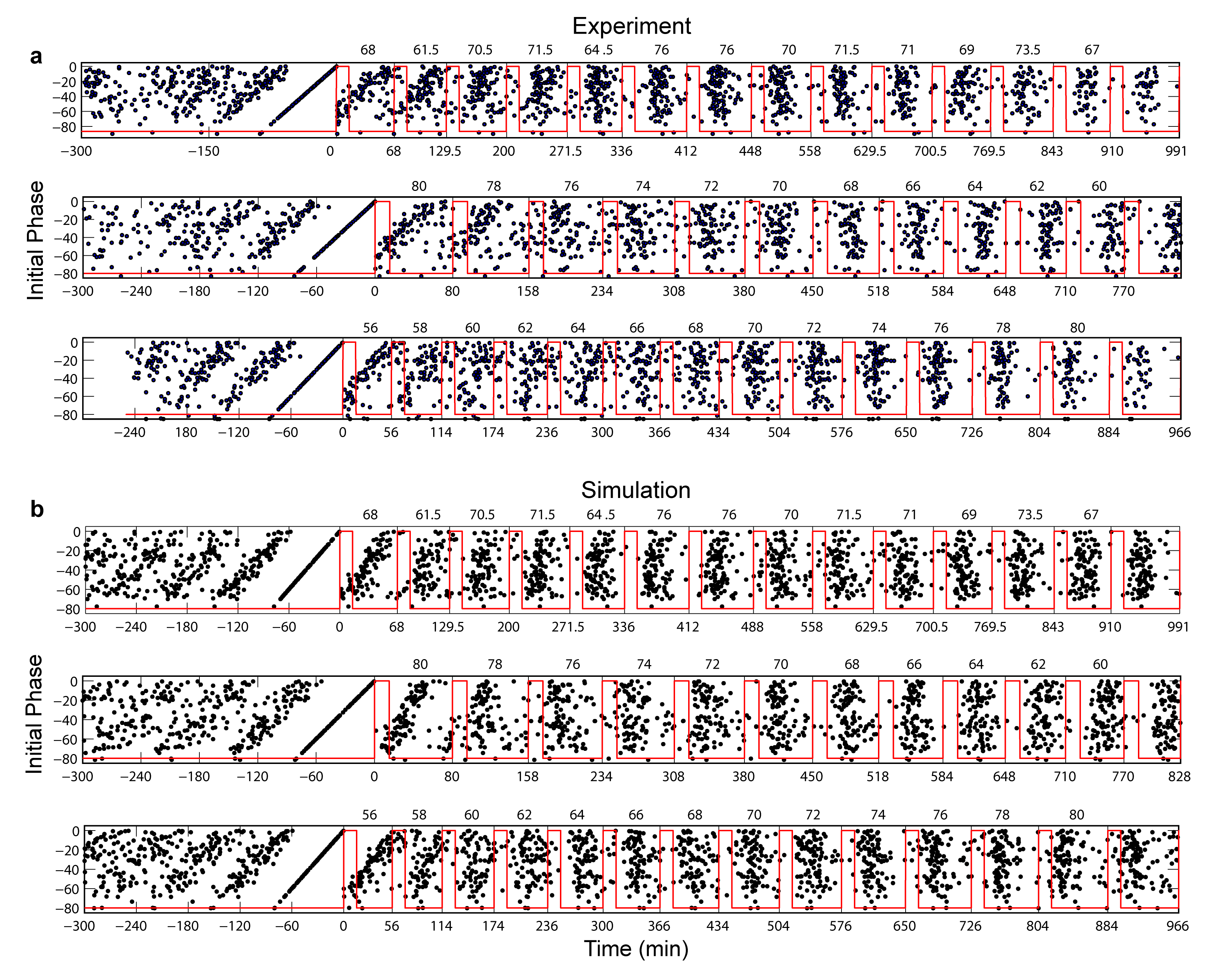
**

**Figure S9.**

**
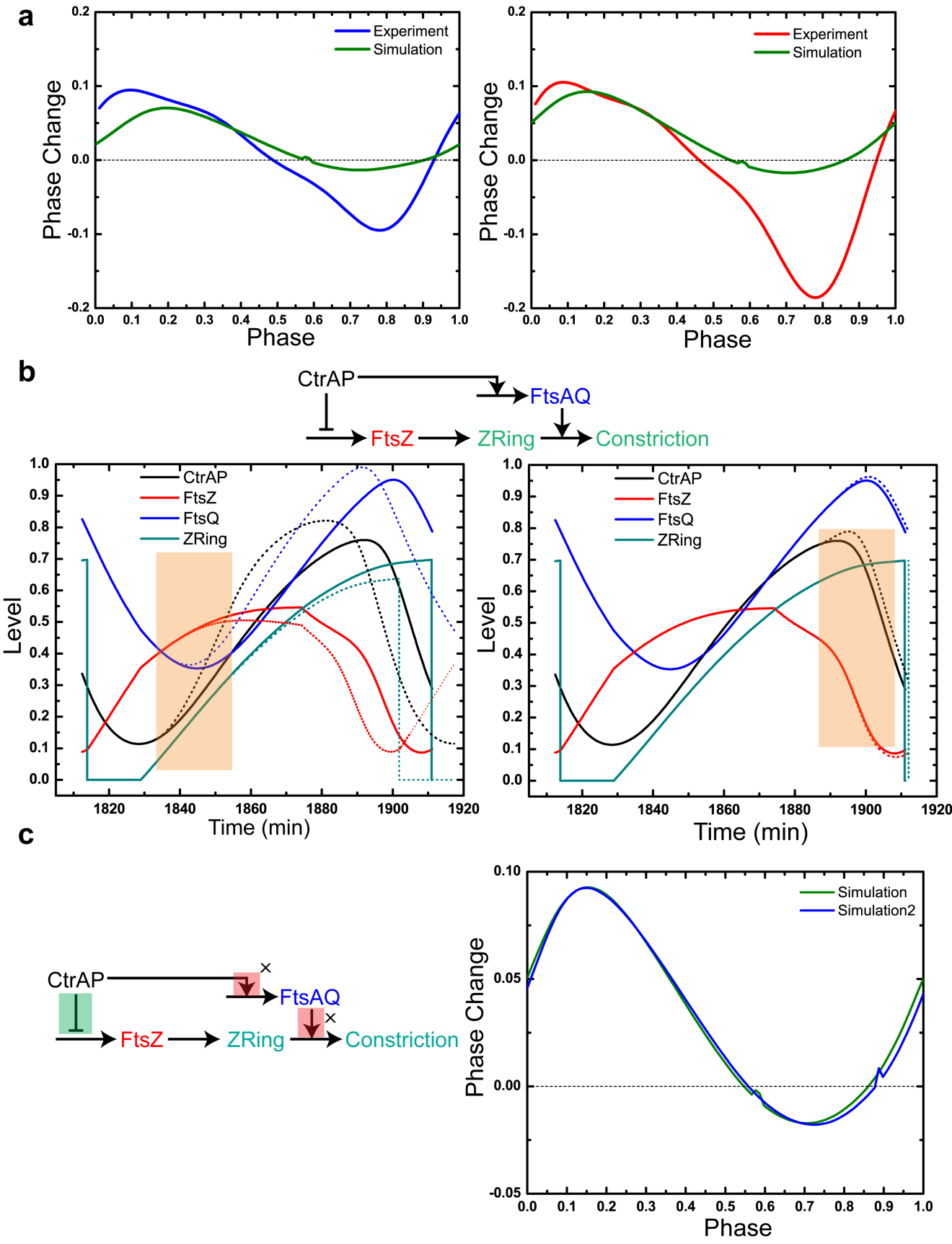
**

**Figure S10.**


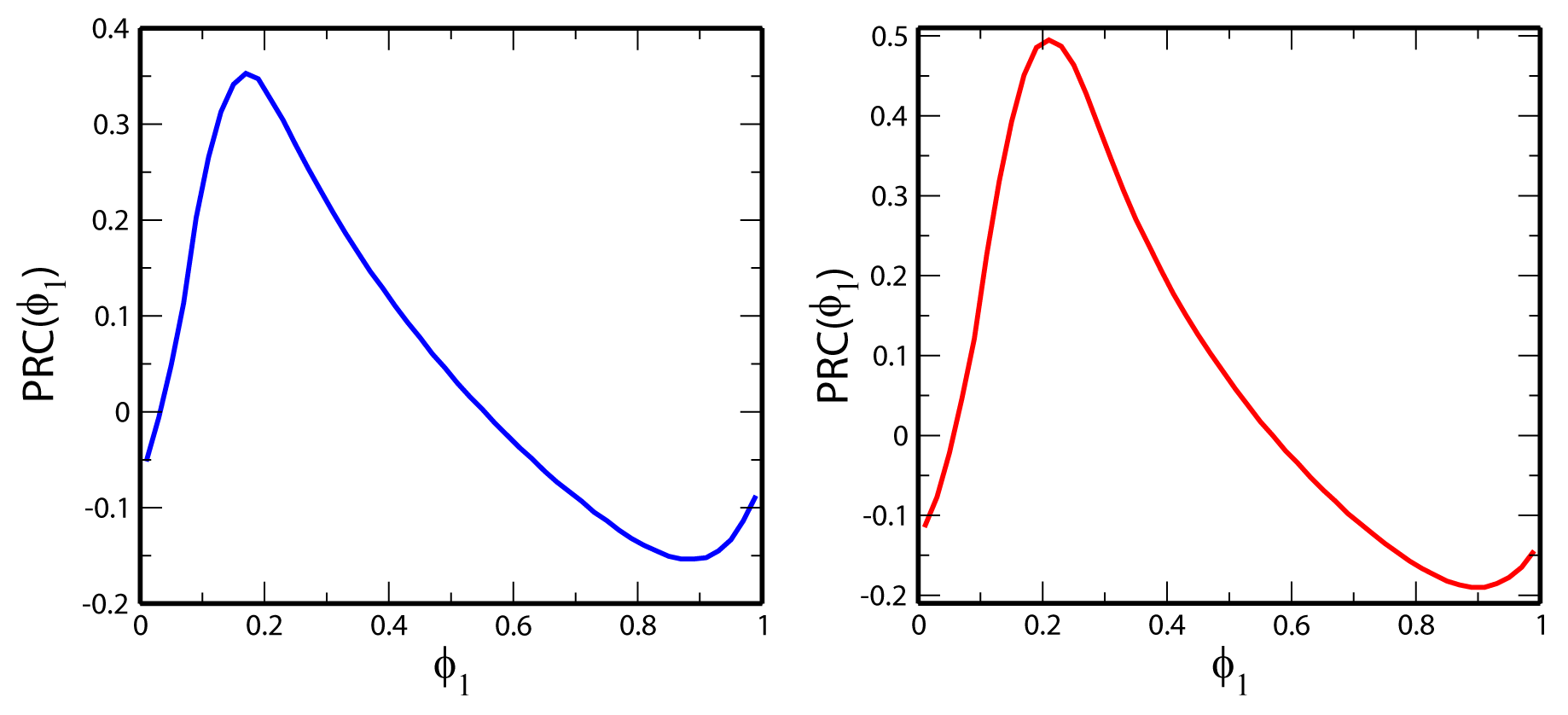


**Figure S11.**

**
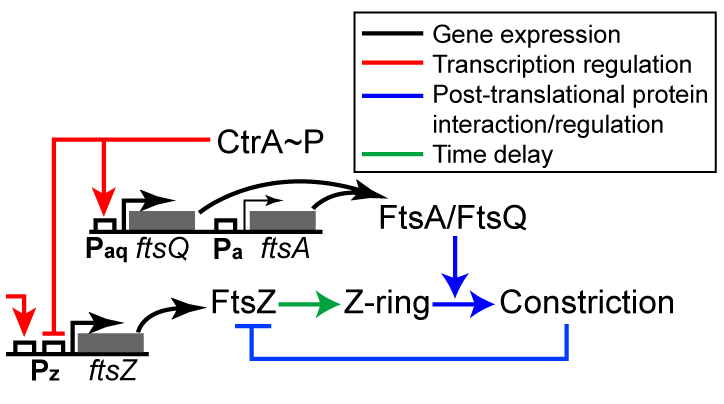
**

**Figure S12.**


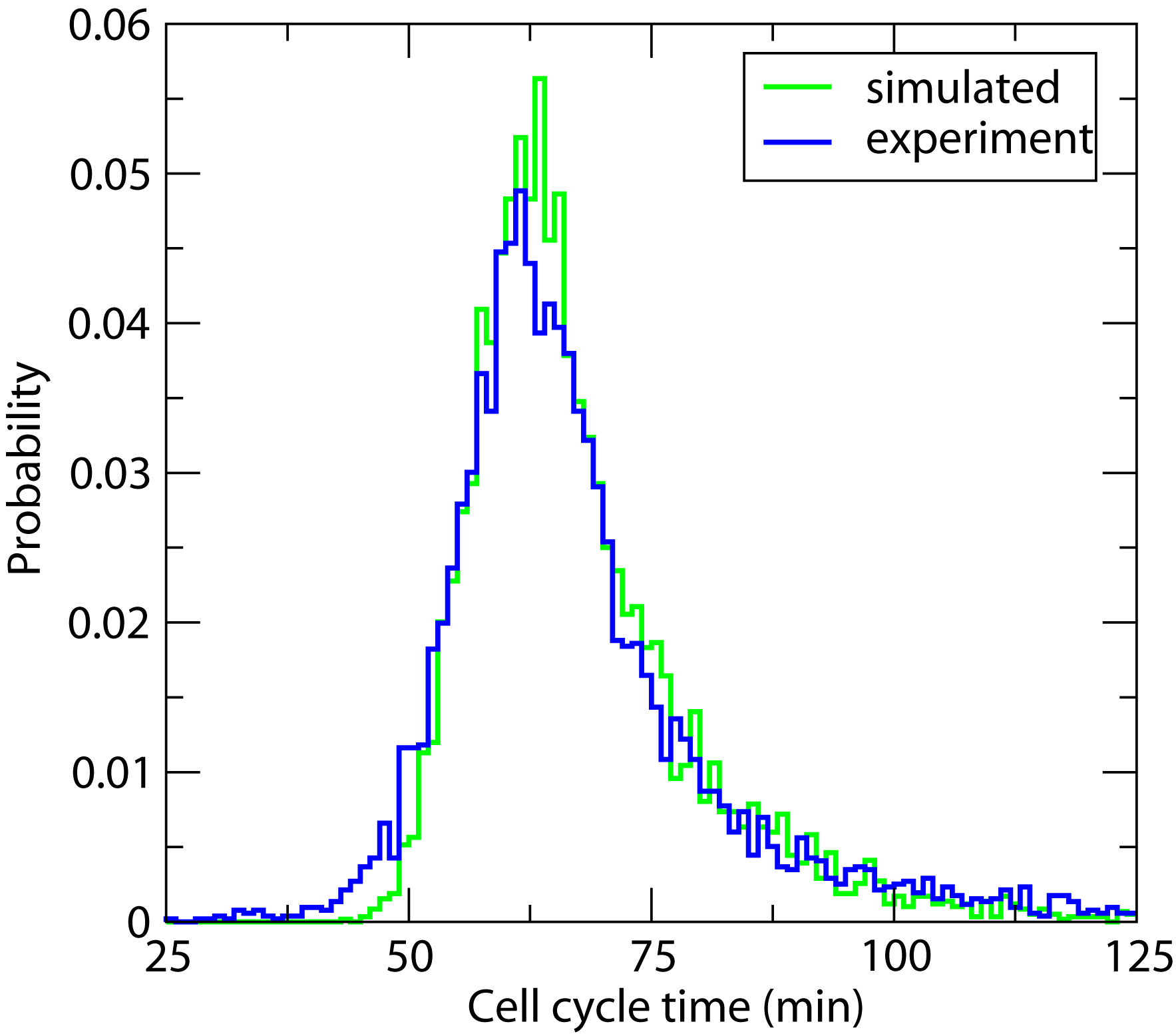


**References for Text S1:**

1. Winfree AT (2001) The geometry of biological time. New York: Springer.

2. Winfree AT (1967) Biological rhythms and the behavior of populations of coupled oscillators. Journal of Theoretical Biology 16: 15-42.

3. Izhikevich EM (2007) Dynamical systems in neuroscience : the geometry of excitability and bursting. Cambridge, Mass.: MIT Press. xvi, 441 p. p.

4. Sancho JM, Miguel MS, Katz SL, Gunton JD (1982) Analytical and numerical studies of multiplicative noise. Physical Review A 26: 1589.

5. Li S, Brazhnik P, Sobral B, Tyson JJ (2009) Temporal controls of the asymmetric cell division cycle in Caulobacter crescentus. PLoS Comput Biol 5: e1000463.

6. Kelly AJ, Sackett MJ, Din N, Quardokus E, Brun YV (1998) Cell cycle-dependent transcriptional and proteolytic regulation of FtsZ in *Caulobacter*. Genes & Development 12: 880-893.

7. Sackett MJ, Kelly AJ, Brun YV (1998) Ordered expression of ftsQA and ftsZ during the Caulobacter crescentus cell cycle. Molecular Microbiology 28: 421-434.

8. Martin ME, Trimble MJ, Brun YV (2004) Cell cycle-dependent abundance, stability and localization of FtsA and FtsQ in Caulobacter crescentus. Molecular Microbiology 54: 60-74.

9. Kuramoto Y (1984) Chemical Oscillations, Waves, and Turbulence. Berlin: Springer.

10. Lin Y, Crosson S, Scherer NF (2010) Single-gene tuning of Caulobacter cell cycle period and noise, swarming motility, and surface adhesion. Molecular Systems Biology 6: 445.
